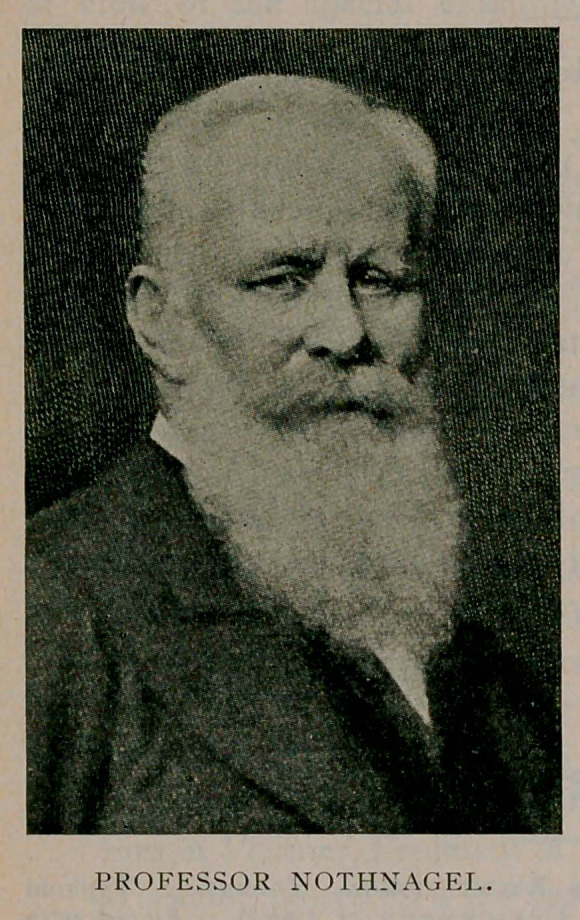# Book Reviews

**Published:** 1902-06

**Authors:** 


### BOOK REVIEWS.

AMERICAN EDITION OF NOTHNAGEL'S ENCYCLOPEDIA. Volume I. Typhoid and Typhus Fevers. By DR. H. CURSCHMANN, of Leipzig. Edited, with additions, by William Osler, M. D , Professor of the Principles and Practice of Medicine, Johns Hopkins University. Octavo of 646 pages. Illustrated; including a number of temperature charts and 2 full-page colored plates.

Volume II. Variola (including Vaccination), by Dr. H. Immermann, of Basle. Varicella, by Dr. Th. von Jürgensen, of Tübingen. Cholera Asiatic and Cholera Nostras, by Dr. C. Liebermeister, of Tübingen. Erysipelas and Erysipeloid, by Dr. H. Lenhartz, of Hamburg. Whooping Cough and Hay Fever, by Dr. G. Sticker, of Giessen. Edited, with additions, by Sir J. W. Moore, B. A., M. D., F. R. C. P. I., Professor of the Practice of Medicine, Royal College of Surgeons, Ireland. Octavo volume of 682 pages. Illustrated. Philadelphia and London: W. B. Saunders & Co 1902. [Price, cloth, $5.00 net; half-morocco, $6.00 net.]

Nothnagel's Encyclopedia will be given a cordial welcome by every progressive physician, but especially by those unfamiliar with the German language, will this American edition be eagerly sought and read. The first volume will prove of special interest because it deals with typhoid fever in an exhaustive manner, bringing its scientific observations down to the latest day.

The etiology of the disease is accorded about 60 pages, in which is contrasted the early opinions with those prevailing at the present time. As a conclusion the assertion is made that the development of typhoid is dependent upon lesions resulting from the invasion of the Eberth-Gaffky bacillus, and that the typhoid bacillus is not identical with the bacillus coli. The section on pathology is interesting, confirming as it does the observations that the chief manifestation of the disease is in the jejunum and downward, but detailing all collateral lesions or alterations that occur in other organs. Of particular moment is the changed elements of the blood, Thayer's work having been utilised in this relation.

PROFESSOR NOTHNAGEL.

The section devoted to the consideration of symptoms and complications is naturally the largest, covering upward of 160 pages and is of absorbing interest. Here the clinician will find every symptom and complication that has yet been observed, carefully described and its due weight given. The surgery of typhoid fever, interest in which was revived by Keen's contribution to the subject, has been dealt with most satisfactorily; indeed, Keen's work has been drawn upon for much material in revising this part of the book. Recrudescences of the disease are believed to be due to a revival of the primary processes rather than a renewal of the infection,—an opinion that appears to be pretty definitely settled at the present time. Perforative peritonitis is always to be dreaded as a sequel of typhoid fever, and much light is thrown upon this portion of the subject by the additions which the American edition contains.

It is conceded by the author that immunity usually is entailed by an attack of typhoid fever, though there are exceptions, and as many as three or four recurrences of the disease in the same individual have been noted. In the diagnosis, clinical investigation is placed in advance of the bacteriologic methods, the latter not yet having reached that perfection which is desirable. Serum-diagnosis is given the credit of being a great aid, and Pfeiffer, Gruber, and Widal are given due acknowledgement for their labors.

In the treatment, diet, environment and hygiene are made to play a far more important rôle than drugs. Hydrotherapy, however, is given a prominent place in the management and antipyretics are used in appropriate conditions, though with a sparing hand. The whole area of typhoid fever is covered in a skillful and scientific manner and the monograph cannot fail to prove of valuable aid to every one called upon to deal with the disease.

The remainder of this volume, about 150 pages, is set apart to the consideration of typhus fever and is full of interest to the teacher of medicine. It is a disease so rarely met with in this country that it has ceased to attract the attention of the everyday practitioner.

The second volume considers the acute infectious exanthemata, as well as cholera, erysipelas, whooping cough, and hay fever. Hence, it becomes of great value to the health authorities, who administer the great trusts of preventive medicine in the large centers of population. Perhaps at this time the chief interest will group around the chapter on vaccination, in view of the fact that this most potent prevention to the spread of smallpox is being attacked by the ignorant, the superstitious and the super-refined.

It will be pertinent, perhaps, to quote from the closing remarks of this section the following paragraph:

> Scruples on the part of the state against compulsory vaccination must be designated as weak, since vaccination is not only useful to the individual, but indirectly protects the whole community. Moreover, compulsory vaccination of children is not only a desideratum, but an ethical duty, since children as yet without the power of deciding for themselves should not be given over arbitrarily to their elders and thereby eventually become the prey of variola.

This succinct statement of the duty of the state and the ethics of the subject should, alone, cause the opponents of vaccination to pause in their mad antagonism, and ask themselves what right they have to take a stand so dangerous to the community.

Erysipelas is a disease too little understood by the average medical practitioner, and may be studied here with profit. The pathology of this peculiar affection is set forth with surprising intelligence and interest. Whooping cough and hay fever are described in the final chapters, and the index and references serve to complete a book of remarkable utility.

---

A MANUAL OF CLINICAL LABORATORY METHODS. By JOHN BENJAMIN NICHOLS, M. D., in Charge of the Clinical Laboratory of Garfield Hospital; Hematologist to Columbian Hospital; Professor of Normal Histology in the Medical Department of Columbian University, Washington, D. C. Illustrated. New York: William Wood & Co. 1901.

This work is prepared to meet a necessity growing out of the prevailing methods of clinical diagnosis. It first describes the necessary equipment for such investigations and then proceeds to instruct in the examination of the fluids and solids of the body, beginning with the blood. Nichols's technic is simple but thorough, and is ample for the practical purposes intended.

The stomach is next taken up, its character first described and then its contents examined during fasting and after test meals; its washings, and vomitus are dealt with; and, finally, its motor and absorptive powers are considered. The feces and intestinal discharges are examined with precision; then the sputum and the urine are considered with much detail. The section on examination of the urine is one of the best we have seen and should be carefully studied.

The chapters on the miscellaneous secretions and body fluids, and the pathologic fluids are full of interest, as are those, also, upon parasites, clinical bacteriology and autopsies. An excellent index closes the volume, which contains forty-two illustrations. It is a book full of useful information and adds to the literature of medicine in a substantial manner. It helps to make the practising physician more accurate by stimulating him to research work in his own cases, instead of sending to a distance, which entails delay and divides his income. Furthermore, it generates a scientific esprit de corps in the medical profession to study such a book, and then to proceed to put the knowledge which it inculcates into practical application.

---

PROGRESSIVE MEDICINE, Vol. I., 1902. A Quarterly Digest of Advances, Discoveries and Improvements in the Medical and Surgical Sciences. Edited by HOBART AMORY HARE, M. D., Professor of Therapeutics and Materia Medica in the Jefferson Medical College of Philadelphia. Octavo, 452 pages, 5 illustrations. Philadelphia and New York: Lea Brothers & Co. 1902. [Per annum, in four cloth-bound volumes, $10.00; per volume, $2.50, by express prepaid to any address.]

This volume appears with its customary promptitude and is prepared with the usual skill and care of its accomplished editor. Its contents includes surgery of the head, neck, and chest by Charles H. Frazier; infectious diseases, including acute rheumatism, croupus pneumonia, and influenza, by Frederick A. Packard; diseases of children, by Floyd M. Crandall; pathology, by Ludvig Hektoen; laryngology and rhinology, by St. Clair Thompson (of London); otology, by Robert L. Randolph, and an admirable index.

It is an excellent selection of the valuable literature that has appeared recently on these subjects, and makes an important addition to the working library of the busy physician.

---

TRANSACTIONS of the American Surgical Association. Volume the Nineteenth. For the year 1901. Edited by RICHARD H. HERTER, M. D., Recorder of the Association. Pp. xxviii.—514. Philadelphia: William J. Dornan, Printer. 1901.

This book contains a record of the work done by this distinguished association at its annual meeting, May 7-9, 1901, which we believe was held at Baltimore, though there is nothing in the volume to indicate the fact. The association was presided over by Dr. Roswell Park, of Buffalo, who chose for the subject of his presidential address, The recent Buffalo investigations regarding the nature of cancer, a topic involving questions of interest at this time in all medical centers, especially where laboratory investigations are pursued. A group of papers pertaining to cancer followed the address, by Thomas S. Cullen, W. S. Halsted, William B. Coley and Joseph D. Bryant, which taken together, make interesting material for the reader who is keeping in touch with the literature of the subject.

There are other valuable surgical papers in this volume which is one of the best the association has issued, and every surgeon will enjoy reading it.

## Figures and Tables

**Figure f1:**